# Focal adhesion-mediated directional cell migration guided by gradient-stretched substrate

**DOI:** 10.1016/j.isci.2024.110446

**Published:** 2024-07-04

**Authors:** Zijia Chen, Xiaoning Han, Bo Che, Huiping Feng, Yue Zhou, Linhong Deng, Xiang Wang

**Affiliations:** 1 Institute of Biomedical Engineering and Health Sciences, Changzhou, Jiangsu, China; 2 School of Medical and Health Engineering, Changzhou, Jiangsu, China; 3 School of Pharmacy, Changzhou University, Changzhou, Jiangsu, China

**Keywords:** Haematology, Molecular biology, Transcriptomics

## Abstract

Soft tissues experience strain under mechanical stresses, storing energy as residual stresses and strain energy. However, the specific impact of such strain on cell migration and its molecular mechanisms remains unclear. In this study, we investigated this by using polydimethylsiloxane (PDMS) membranes with varying prestrain levels but constant stiffness to mimic tissue-like conditions. Results showed that higher prestrain levels enhanced 3T3 fibroblast adhesion and reduced filopodia formation. Elevated prestrain also increased integrin and vinculin expression, which was associated with lower cell migration rates. Notably, both 3T3 fibroblasts and primary rat airway smooth muscle cells migrated faster toward higher prestrain areas on substrates with strain gradients. Knockdown of integrin or vinculin inhibited 3T3 cell migration directionality, highlighting their critical role. This research reveals a mechanobiological pathway where strain gradients direct cell migration, providing insight into a common mechanotransduction pathway influencing cellular responses to both stiffness and strain-related mechanical cues.

## Highlights

• Prestrained PDMS substrates enhance cell adhesion and reduce filopodia formation
• Prestrained substrates inhibit cell migration speed
• Gradient-prestrained substrates induce cell migration toward high strain areas
• Integrin and vinculin are crucial for gradient-strain guided directional migration

## Graphical abstract

**Figure:**
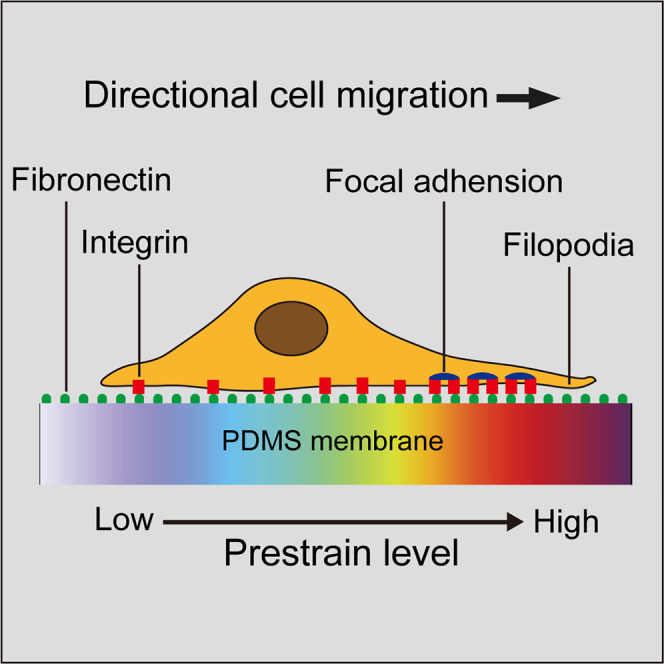

## Introduction

Cell migration is a crucial physiological process in embryonic development, tissue regeneration, immune responses, and the onset and progression of diseases.^1^ Typically, many cells exhibit directed migration responses to various chemical signals, such as molecular or electrochemical gradients, termed chemotaxis and galvanotaxis, respectively.^2^^,^^3^ Furthermore, certain cell types can sense the stiffness of their surrounding extracellular matrix (ECM) and initiate individual or collective migration from softer to stiffer substrates, a phenomenon known as durotaxis.^4^^,^^5^^,^^6^^,^^7^ Further studies reveal that cells detect ECM stiffness through the engagement of focal adhesions (FAs), comprised of integrins and adaptor proteins, such as vinculin.^8^^,^^9^^,^^10^ Hence, the phenomenon of durotaxis and its underlying molecular mechanism underscores that the mechanical environment functions not merely as a passive supporter and constraint for cells but rather as a dynamic participant regulating cellular behavior.

Recent studies investigating the impact of viscoelasticity substrate on cell adhesion and migration suggest that cells not only perceive the stiffness of ECM but also respond to its dissipation energy,^11^^,^^12^ indicating that the cell recognition of the substrate extends beyond its static hardness. This perception may encompass potential energy, such as residual stresses or strain energy, stored within elastic ECM undergoing deformation by cell contraction or external forces. Our previous study revealed that four-fifths of microtissue stress was residual stresses in collagen-based ECM, accumulated by cellular contraction forces after densification.^13^ *In vivo* observations demonstrate that tissues with significant deformation, such as muscle, cardiac, lung, skin, and tumor tissues, inherently possess stored residual stresses.^14^^,^^15^ This prompts the question: is strain merely a feature generated during tissue growth and remodeling or also an influential factor guiding cell behavior? A study sheds light on this issue, suggesting that prestrained elastic substrates enhance the stiffness of the cytoskeleton and promote cell adhesion, indicating that cells can sense deformation energy associated with the substrate.^16^ Given the established connection between the cellular cytoskeleton and cell migration from previous studies on durotaxis, it is plausible that strain impacts cell migration through mechanical sensors.

To investigate this hypothesis, we fabricated elastic membranes using polydimethylsiloxane (PDMS). Stretching the membrane increases strain energy due to the energy needed for realigning and altering chain configurations, leading to reduced entropy and heightened enthalpy.^17^ Consequently, prestraining the membranes to various extents generates different levels of strain energy, akin to those present in the ECM. Through an analysis of the adhesion and migration of 3T3 fibroblast cultured on these membranes, we found that heightened prestrain in substrates led to enhanced cell adhesion and a decrease in filopodia number, accompanied by a reduction in cell migration speed. Under such conditions, key FA proteins, such as integrin and vinculin, were upregulated. Importantly, we discovered an elevation in cellular polarity and migration velocity toward the direction of high strain area on gradient-stretched substrates. This process could be impeded by the downregulation of integrin and vinculin expression. Therefore, these findings deepen our understanding of how cells perceive and respond to strain-related mechanical cues, elucidating the intricate interplay between cellular mechanics and the microenvironment.

## Results

### Fabrication and characterization of prestrained polydimethylsiloxane membranes

**Figure 1:**
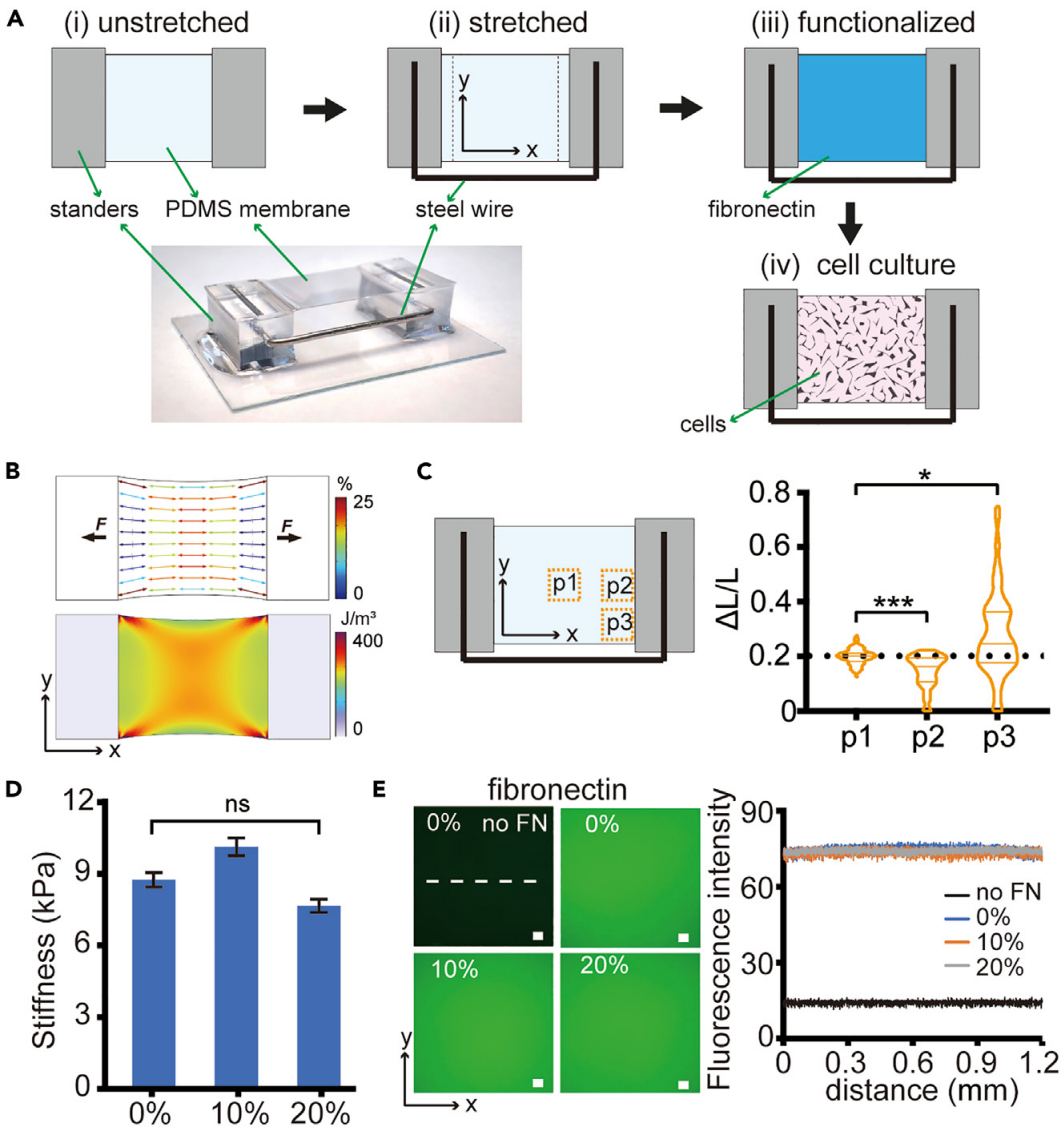
Fabrication and characterization of prestrained PDMS membranes (A) Schematic illustration of device preparation. (B) Simulated distribution of maximum principal strains (upper panel) and strain energy (down panel) in an elastic PDMS membrane prestrained to 20% along the x axis. (C) Relative displacement of microbeads embedded in membranes prestrained to 20% (*n* ≥ 80 beads for each position). Kruskal-Wallis test, ∗*p* < 0.05, ∗∗∗*p* < 0.001. (D) Stiffness of PDMS membranes under 0, 10%, and 20% prestrain conditions. *n* = 75 from 3 independent experiments. Data are presented as means ± SEM. One-way ANOVA test, ns = non-significant. (E) Characterization of fibronectin-coated PDMS membranes by measuring the fluorescence intensity of anti-fibronectin AF-488 staining. Fluorescence intensity profile is drawn along the dashed line in E. Bars = 100 μm.

To investigate the potential impact of strain on cell adhesion and migration, PDMS membranes were subjected to varying levels of stretch. As depicted in Figure 1A, a stretched membrane was upheld by inserting a U-shaped stainless steel wire between the two standers of the membrane. The degree of strain in the PDMS membranes was modulated by adjusting the width of the steel wire. To enhance the biocompatibility of the PDMS substrate for cell culture, fibronectin was covalently linked to the surface after membrane stretching to prevent non-homogeneous coating arising from the membrane strain. Due to the Poisson effect, the distribution of strain is non-uniform in the stretched region, as shown in the simulation result where the area exhibiting relatively uniform strain and strain energy is primarily located in the central region of the membrane (Figure 1B). To validate the simulation, we measured the displacement of microbeads embedded in the PDMS membrane before and after 20% stretching (Figure S1A). The results revealed a uniform 20% strain occurring only in the central region, not in the corners and surrounding areas (Figure 1C). Consequently, only cells located in the central region of membranes were analyzed in the following experiments unless stated otherwise.

Subsequently, the stiffness and protein coating of PDMS membranes with varying prestrain levels were characterized. No significant changes in stiffness were observed among control, 10%, and 20% prestrained membranes (Figures 1D and S1B). This ensured that strain was the sole mechanical variable under consideration in this study. In addition, the static stretch test showed that PDMS membranes retained their elasticity for at least 7 days (Figure S1C), suggesting its capability to preserve strain energy throughout the experimental period. The distribution of fibronectin on substrates was assessed by immunofluorescence staining, which demonstrated uniform staining of fibronectin on the prestained membranes (Figure 1E). Furthermore, functionalized PDMS membranes display surface tension under different strain conditions (Figure S1D). Therefore, we have successfully fabricated fibronectin-coated PDMS membranes encompassing distinct levels of strain with consistent surface stiffness and surface tension.

### Prestrained substrates enhance cell adhesion and inhibit filopodia formation

**Figure 2:**
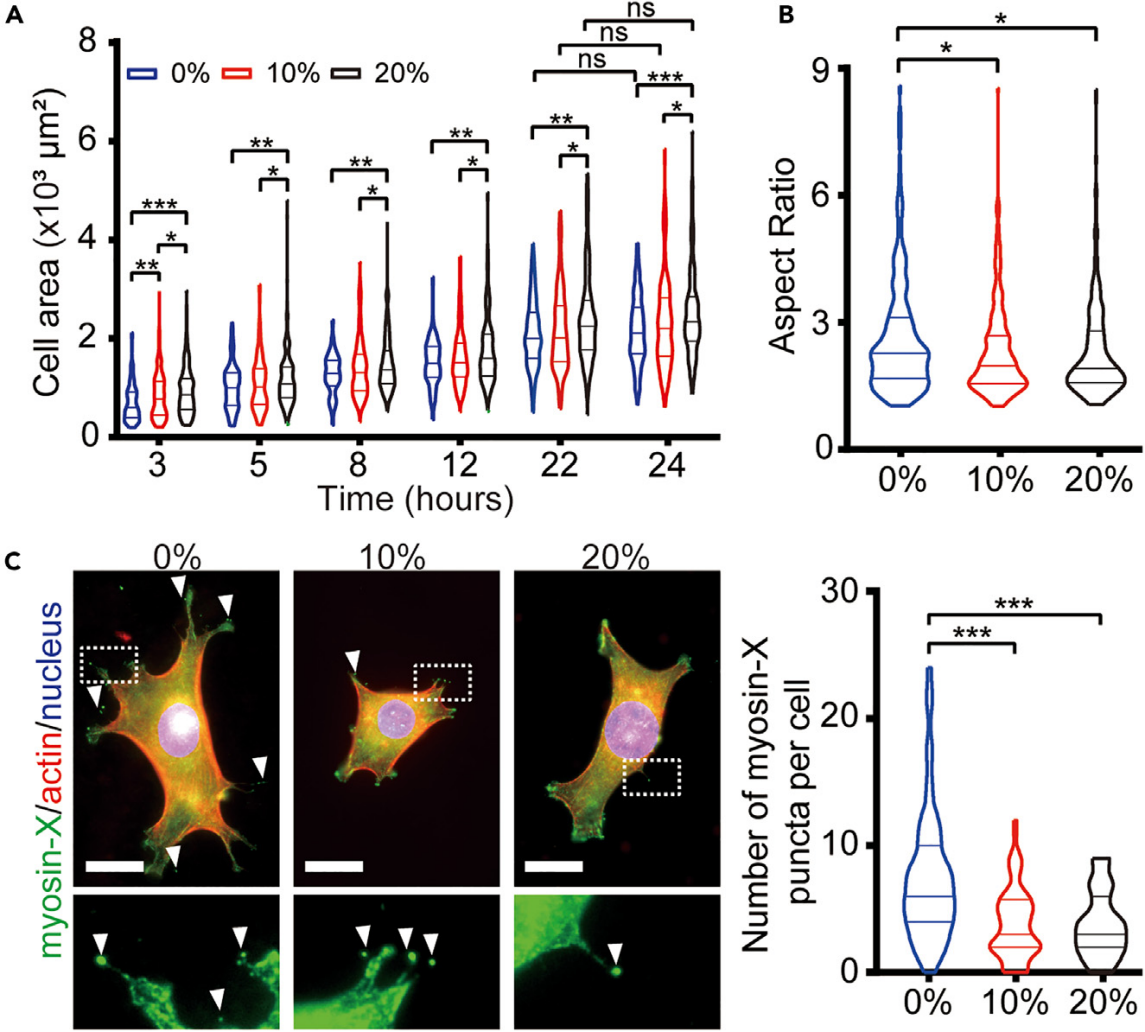
Effects of stretched substrates on cell adhesion (A) Cell spreading areas on different prestrained substrates over 24 h (*n* ≥ 200 cells). Kruskal-Wallis test for groups at the same time points, Mann-Whitney test for groups at different time points, ns = non-significant., ∗*p* < 0.05, ∗∗*p* < 0.01, ∗∗∗*p* < 0.001. (B) Aspect ratio of cells on different prestrained substrates after 24 h of culture (*n* ≥ 200 cells). Kruskal-Wallis test, ∗*p* < 0.05. (C) Evaluation of filopodia number by myosin-X staining (arrowheads) in cells on different prestrained substrates after 24-h culture (*n* ≥ 55 cells). Bar = 20 μm. Kruskal-Wallis test, ∗∗∗*p* < 0.001.

To investigate cell adhesion on substrates with different levels of strain, 3T3 fibroblast cells were seeded onto prestrained membranes by sedimentation and monitored over 24 h. Measurement of cell projection areas revealed a significant increase in cell spreading areas with the degree of prestrain increased at least 24 h after seeding (Figure 2A), which is consistent with the previous finding that higher strain energy is conducive to enhanced cell spreading.^16^ Moreover, the aspect ratio of the cells significantly decreased with an increase in prestrain level (Figure 2B**)**, resulting in a more circular cell shape after 24 h. This suggests that stretched substrate may influence cytoskeleton rearrangements and the polarized states of cells. Subsequently, we examined the formation of filopodia, as they have been widely reported to be associated with cell polarization and migration.^18^^,^^19^ By quantifying the number of the marker protein myosin-X at filopodia tips, we observed a reduced number of myosin-X puncta per cell under higher prestrained conditions (Figure 2C), suggesting that cells under these conditions formed less filopodia. Thus, elevated levels of prestrained substrates lead to increased cell spreading area, a more rounded cell shape, and a concurrent reduction in filopodia assembly.

### Prestrained substrates upregulate integrin and vinculin expression

**Figure 3:**
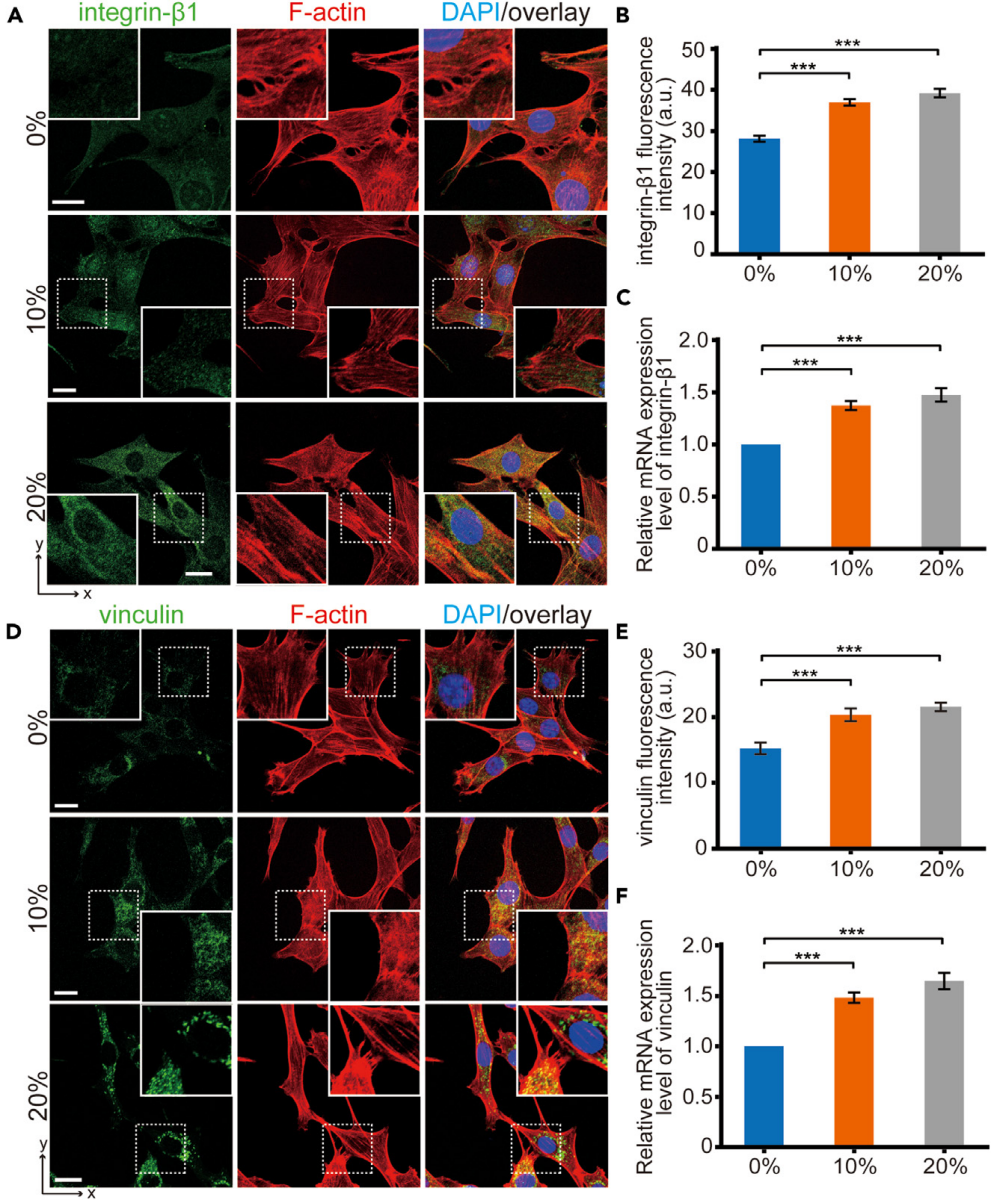
Increased expression of integrin and vinculin in cells on prestrained substrates (A) Representative immunostaining of integrin-β1 images and (B) quantification of fluorescence intensity in cells cultured on different prestrained substrates after 24 h (*n* = 200 cells). (C) Quantification of integrin-β1 mRNA levels after 24 h of culture (*n* = 4 independent experiments). (D) Representative immunostaining of vinculin images and (E) quantification of fluorescence intensity in cells cultured on different prestrained substrates after 24 h (*n* = 200 cells). (F) Quantification of vinculin mRNA levels after 24 h of culture (*n* = 4 independent experiments). Bars = 20 μm. Data are presented as means ± SEM. Kruskal-Wallis test, ∗∗∗*p* < 0.001.

Understanding the molecular mechanisms involved in strain-induced cellular response is crucial. Integrins act as primary force-bearing structures linking the ECM and FAs, playing pivotal roles in the sensing of the mechanical properties of ECM.^20^^,^^21^ In light of this, we examined the expression of integrin-β1 in cells grown on substrates with varying prestrain levels (0%, 10%, and 20%) through immunofluorescence imaging (Figure 3A). The result showed a significant increase in integrin-β1 protein expression under higher prestrain conditions (Figure 3B). This was further validated by Q-PCR analysis of integrin-β1 mRNA expression within the cells (Figure 3C). As a key component of FAs, vinculin serves to link integrins to the actin cytoskeleton.^10^^,^^22^ Thus, we assessed the expression of vinculin within cells on substrates with different prestrain levels. The results demonstrated a significant increase in vinculin protein expression on 20% prestrained substrates (Figures 3D and 3E), which was further confirmed by Q-PCR analysis (Figure 3F). Consequently, elevated levels of strain concurrently upregulate the expression of integrin and vinculin, suggesting that cells may sense and respond to the mechanical strain of the ECM through the interaction and signaling of FAs.

### Prestrained substrates inhibit cell migration

**Figure 4:**
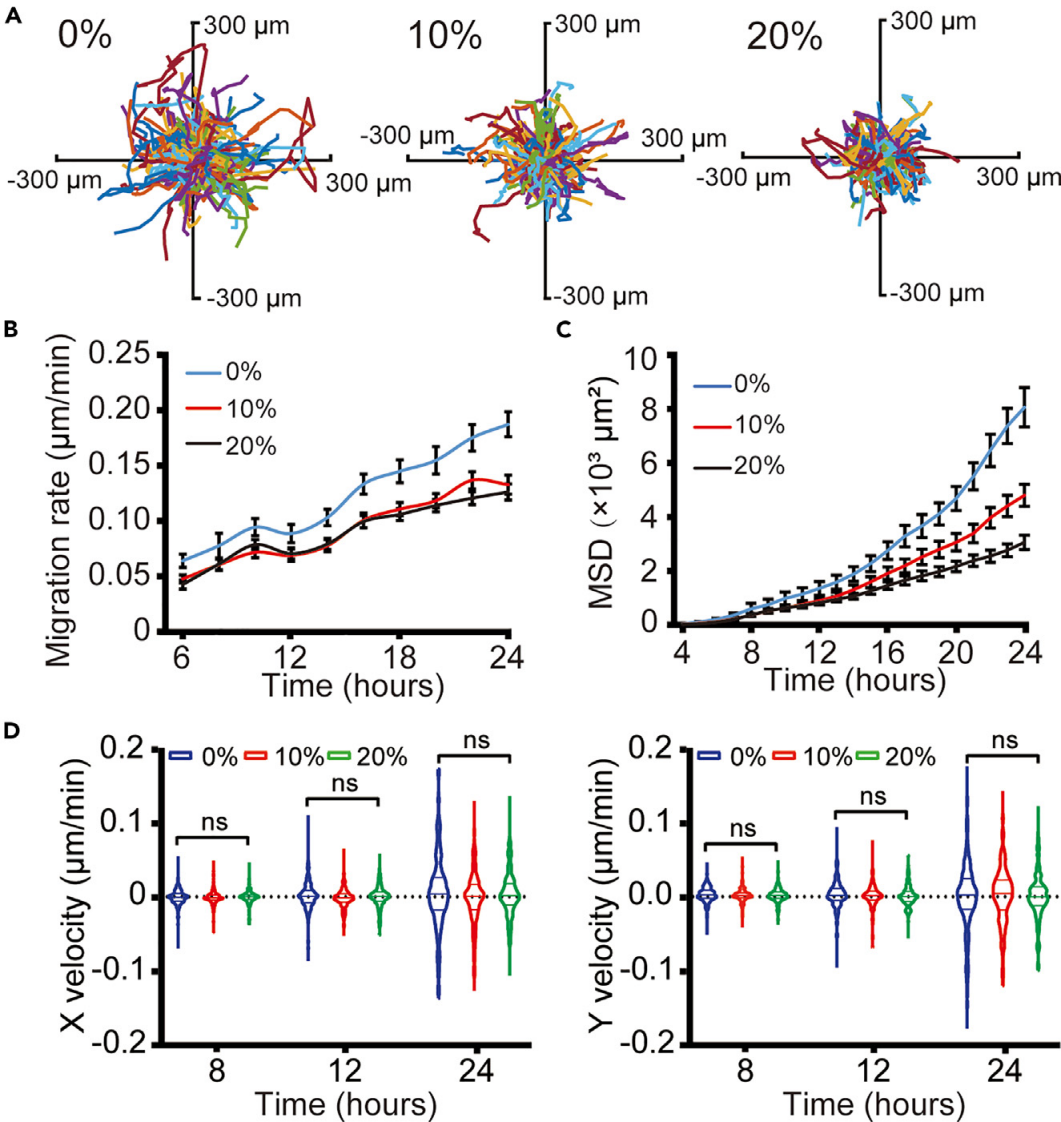
Impact of prestrained substrates on 3T3 cell migration (A) Trajectories of randomly selected migrating cells on different prestrained substrates over 24 h. (B) Migration rates of cells cultured on different prestrained substrates (*n* = 4 independent experiments). Data are presented as means ± SEM. (C) Mean squared displacement (MSD) plotted versus time lag for cells on different prestrained conditions (*n* = 4 independent experiments). Data are presented as means ± SEM. (D) X and Y velocity of cells on different prestrained substrates over 24 h *n* = 298 (0%), 314 (10%) and 315 (20%) cells. Kruskal-Wallis test, ns = non-significant.

To investigate the impact of prestrained substrates on cell migration, time-lapse imaging was used to monitor individual cell movement on PDMS membranes with 0, 10%, and 20% strain. The result revealed that 3T3 cells exhibited a reduced range of movement on prestrained substrates compared to the condition without prestrain (Figure 4A). Statistical analysis indicated that on prestrained substrates, the cell migration rate was significantly lower than that on unstrained substrates (Figure 4B). Consistent findings were obtained through mean square displacement (MSD) analysis, demonstrating that 3T3 cells cultured on unstrained substrate exhibited significantly faster motility compared to cells cultured on substrates with 10% and 20% strain (Figure 4C). To study whether the migration direction is also influenced by the prestrained substrate, we calculated the migration velocity in the horizontal (x axis) and vertical (y axis) directions relative to the strain. There were no significant differences in both directions (Figure 4D), suggesting that prestrained substrates impact the speed of cell migration while having no effect on the direction of cell migration. To further investigate whether the above observation was a common phenomenon, we performed the same experiments using airway smooth muscle (ASM) cells. Similar to 3T3 cells, the migration activity of ASM cells was less on prestrained substrates than on unstrained substrates (Figures S2A–S2C), suggesting that both cell types respond similarly to mechanical cues from the prestrained substrates.

### Gradient-prestrained substrates induce directional cell migration

**Figure 5:**
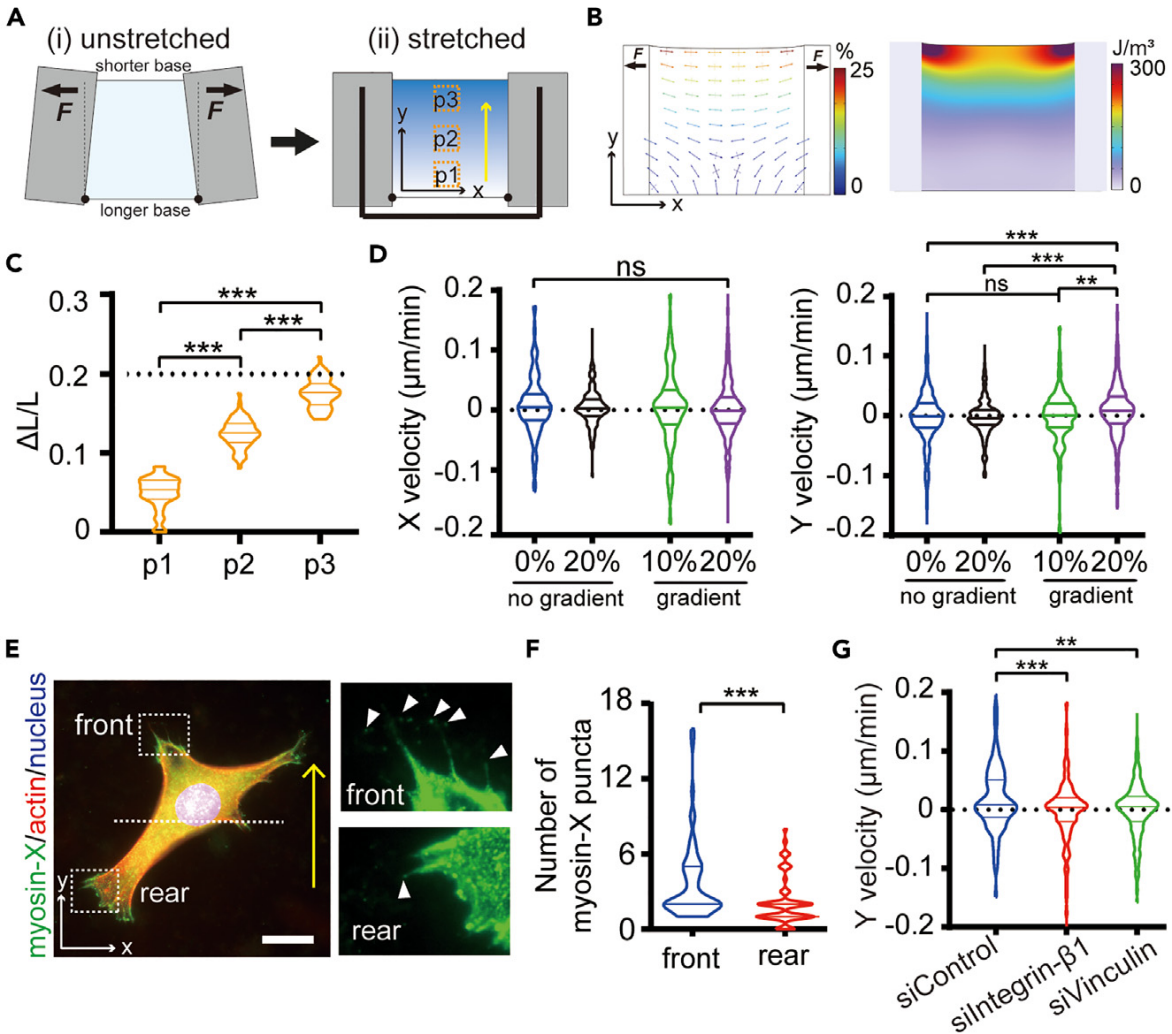
Effects of gradient-prestrained substrates on directional cell migration (A) Schematic illustration of the preparation of gradient-prestrained substrate device. The yellow arrow denotes regions with elevated prestrain. (B) Simulated distribution of maximum principal strains (left panel) and strain energy (right panel) in an elastic PDMS membrane prestrained to 20% along the shorter base. (C) Relative displacement of microbeads embedded in different regions of membranes shown in (A) after gradient stretching to 20% (*n* = 50 beads). Kruskal-Wallis test, ∗∗∗*p* < 0.001. (D) X and Y velocity of 3T3 cells on normal prestrained (0, 20%) and gradient prestrained (10%, 20%) substrates (*n* ≥ 200 cells). Kruskal-Wallis test, ns = non-significant, ∗∗*p* < 0.01, ∗∗∗*p* < 0.001. (E) Polarized distribution of filopodia along the gradient direction (arrow), as indicated by myosin-X staining (arrowheads). Bar = 20 μm. (F) Quantification of myosin-X puncta in 3T3 cell front (high prestrained direction) and rear (low prestrained direction) regions (*n* = 56 cells). Mann-Whitney test, ∗∗∗*p* < 0.001. (G) Inhibition of directional 3T3 cell migration (control, *n* = 203 cells) with the knockdown of integrin-β1 (*n* = 394 cells) or vinculin (*n* = 408 cells). Kruskal-Wallis test, ∗∗*p* < 0.01, ∗∗∗*p* < 0.001.

The distinct migration behaviors exhibited on prestrained substrates raised the question of whether cells can sense gradient changes in substrate strain on the same surface. To investigate this hypothesis, we fabricated gradient-prestrained PDMS membranes by applying tensile forces to the short base of a trapezoidal membrane, transforming it into a square shape (Figure 5A). As demonstrated by the simulation result (Figure 5B), the strain and strain energy in the region close to the short base is higher than in the region on the opposite side. Consequently, a strain gradient was generated perpendicular to the direction of tensile forces. Measurement of the positions of microbeads within the membranes both before and after stretching demonstrates a progressively increased displacement along the y axis direction (Figure 5C). Upon seeding 3T3 or ASM cells onto substrates featuring different gradient prestrain (ranging from 0% to 10% or 20%) and culturing them for 24 h, no significant differences in cell migration velocity were observed in the x axis (direction perpendicular to the gradient) (Figures 5D and S2D**, left**). Interestingly, on the 20% gradient-prestrained substrate, cells exhibited a higher migration velocity toward the high prestrain region compared to the condition without a gradient (Figures 5D and S2D**, right**). Nonetheless, this phenomenon was not observed on 10% gradient-prestrained substrates, suggesting that 3T3 and ASM cells are capable of sensing and responding to specific levels of gradient changes in substrate strain.

To investigate alterations in cell polarity during directional migration, we distinguished filopodia assembled at the cell front (toward the high prestrain direction) from those at the rear (toward the low prestrain direction). Subsequently, we quantified the number of myosin-X puncta in the front and rear regions of randomly selected 3T3 cells cultured on 20% gradient-prestrained substrates (Figure 5E). The results revealed a significantly higher number of puncta in the front region compared to the rear region of cells (Figure 5F). Therefore, it is evident that gradient strain in the matrix influences filopodia distribution, consequently impacting cell polarity. Moreover, to elucidate the involvement of FAs molecules in perceiving these gradient mechanical cues, we employed siRNA knockdown of integrin-β1 and vinculin expression in 3T3 cells (Figure S3). The knockdown of these two molecules resulted in a significant decrease in cell migration velocity toward the high prestrain direction (Figure 5G). These findings suggest a dependence on focal adhesions in enabling directional cell migration to respond to gradient strain.

## Discussion

When tissue is subjected to forces, ECM undergoes deformation, leading to the storage of strain energy within the tissue. To investigate the effects of strain on fibroblast behavior and the underlying molecular determinants, we cultured 3T3 cells on prestrained elastic membranes with an equivalent stiffness to unstrained membranes. By comparing the morphological features of cells on prestrained and unstrained substrates, we observed an increased cell adhesion area on prestrained substrates. Similarly, 3T3 cells exhibit increased spreading areas on stiffer substrates,^23^^,^^24^^,^^25^ possibly due to the molecular mechanism where forces develop faster on stiff ECM. This leads to the stabilization of FAs and subsequently increases cell-matrix interfacial tension, which is a critical factor contributing to enhanced cell wetting.^26^^,^^27^ Additionally, we discovered that cells on prestrained substrates formed less filopodia, as evidenced by myosin-X labeling. Likewise, human lung cancer cells growing on stiffer substrates have less filopodia.^28^ Taken together, our results suggest that fibroblasts display similar adhesion behaviors on prestrained substrates and stiff substrates.

Besides influencing cell adhesion, our results revealed a significant decrease in cell migration speed on prestrained substrates compared to unstrained substrates, resembling the observed phenomenon of lower migration speed of 3T3 cells on stiff substrates compared to soft substrates.^29^ We also found that the migration direction on prestrained substrates remained unaffected by the stretch direction, which may be due to the directionless nature of strain energy. Nevertheless, in situations with uneven strain or stress distribution, directionality may occur along the gradient of strain. Indeed, we identified a durotaxis-like phenomenon: the migration velocity of 3T3 cells toward high-strained regions was higher than that toward low-strained regions. It’s noteworthy that such directional cell migration is dependent on the steepness of the strain gradient, similar to durotaxis, which relies on the steepness of the stiffness gradient.^4^ Directional cell migration guided by strain gradients may play unique roles in tissue development and wound healing. For example, in tissue morphogenesis where residual stress accumulates,^30^ strain gradients may contribute to the alignment and migration of cells, aiding in the formation of complex tissue architectures. In the context of wound healing, strain arises at the wound site.^31^^,^^32^ The strain gradient may promote cells in the surrounding tissue to migrate toward the site to facilitate the closure of the wound.

Our research indicates that the morphology and migration of 3T3 cells are responsive to the level of strain in the substrates, akin to the impact of different stiffness levels on cell behaviors. At the molecular level, FAs at the ECM interface are directly involved in sensing mechanical properties, including substrate rigidity.^33^ For example, 3T3 cells exhibit elevated integrin expression on stiff substrates compared to soft ones.^23^ Interestingly, our study demonstrated an up-regulation in the expression of integrins and vinculin when cells were cultured on prestrained substrates. Importantly, inhibiting integrins and vinculin by siRNA significantly impeded directional cell migration on gradient-prestrained substrates, underscoring the crucial role of FAs in sensing strain. This suggests a common mechanosensory mechanism in cells for detecting both strain and stiffness associated with the substrate. A recent theory, grounded in the existing clutch model, proposes an explanation for how cells respond to strain caused by deformation.^34^ According to this theory, an additional spring is integrated to represent ECM strain when deformed before interacting with the cell’s molecular clutches. This modification enhances the mechanical transduction from the ECM to the cytoskeleton through FAs, producing effects similar to those observed on a stiffer substrate. Materials such as PDMS or biological tissues that undergo large-scale deformations exhibit nonlinear elasticity, influencing mechanical variables such as stress distribution and strain energy density. This complexity presents significant difficulty for the theoretical computation of biomechanical interactions between cells and the ECM under these conditions. Furthermore, it is challenging to discern the effects of strain from stiffness within tissues, as many ECM strain patterns often coincide with changes in matrix stiffness.^13^^,^^35^ Therefore, future research should focus on elucidating how cell-directed migration is influenced by the simultaneous presence of stiffness and strain gradients, to better mimic the complex mechanical microenvironment found *in vivo*.

In summary, our study demonstrated that manipulating the strain of substrates under a consistent stiffness condition influences cell adhesion and prompts directional migration, mediated by the mechanosensitive molecules integrin and vinculin. These cells may use the mechanism of mechanosensing stiffness to respond to strain energy traits of substrates. Understanding the biological significance of strain gradient-guided directional cell migration offers valuable insights with implications for tissue development and wound healing, as well as tissue engineering and regenerative medicine.

### Limitations of study

Although this study reveals that the strain of substrates influences cell adhesion and migration, there are several limitations. First, the *in vitro* conditions of prestrained PDMS membranes may not fully replicate the complex mechanical microenvironment present *in vivo*, potentially limiting the applicability of our results to physiological contexts. Additionally, the use of 3T3 fibroblasts and ASM cells may not capture the diverse responses of other cell types to strain gradients. Moreover, the study primarily focuses on short-term cellular responses, and long-term effects of substrate strain on cell migration remain unexplored. Finally, while the study identifies the involvement of integrin and vinculin in strain sensing, it does not fully elucidate the downstream signaling pathways and molecular mechanisms underlying this process, necessitating further investigation to achieve a comprehensive understanding.

## STAR★Methods

### Key resources table

**Table:** 

REAGENT or RESOURCE	SOURCE	IDENTIFIER
**Antibodies**		

Anti-MYO10 antibody	Sigma-Aldrich, USA	Cat# HPA024223; RRID:AB_1854248
Anti-integrin-β1	Sigma-Aldrich, USA	Cat# ZRB1230; RRID:AB_3068459
Anti-vinculin	Abcam, USA	Cat# Ab129002; RRID:AB_11144129
Goat anti-Rabbit IgG	Thermo Fisher Scientific, USA	Cat# A-11008; RRID:AB_143165

**Biological samples**		

NIH/3T3 Fibroblast Cells	American Type Cell Culture, USA	Cat# CRL-1658
Primary Rat Airway Smooth Muscle Cells	BeNa Culture Collection, China	Cat# 235298

**Chemicals, peptides, and recombinant proteins**		

Sylgard 184 Silicone Elastomer Kit	Dow Corning, USA	Cat# 1673921
Baysilone-Paste	GE Bayer	Cat# V000332
3-Aminopropyltriethoxysilane (APTES)	Sigma-Aldrich, USA	Cat# 440140
Glutaraldehyde 25% Solution	Aladdin, China	Cat# G105908
Fibronectin	Thermo Fisher Scientific, USA	Cat# 33016015
Paraformaldehyde 4% Solution	Sigma-Aldrich, USA	Cat# 158127
Triton X-100	Sigma-Aldrich, USA	Cat# T8787
Bovine Serum Albumin (BSA)	Roche, Switzerland	Cat# 9048-46-8
Phalloidin, FITC-conjugated	Yeasen Biotech, China	Cat# F1022
DAPI	Sigma-Aldrich, USA	Cat# D9542
Polystyrene microspheres 20μm	Sigma-Aldrich, USA	Cat# 87896
RIPA lysis buffer	Beyotime, China	Cat# P0013B
Phosphatase inhibitor cocktails	Beyotime, China	Cat# P1082
SDS-PAGE protein loading buffer	Beyotime, China	Cat# P0015
180 kDa pre-stained protein marker	Vazyme, China	Cat# MP102

**Experimental models: Cell lines**		

NIH/3T3 Fibroblast Cells	American Type Cell Culture, USA	Cat# CRL-1658
Primary Rat Airway Smooth Muscle Cells	BeNa Culture Collection, China	Cat# 235298

**Oligonucleotides**		

siIntegrin sequence: GGACGAAAGUGUUCCAACA	RiboBio, China	N/A
siVinculin sequence: GGUAGUGGAAACUAUGGAA	RiboBio, China	N/A
Primers for β-actin, see Table S1	This paper	N/A
Primers for integrin-β1, see Table S1	This paper	N/A
Primers for vinculin, see Table S1	This paper	N/A

**Software and algorithms**		

COMSOL Multiphysics 6.2	COMSOL, Sweden	www.comsol.com/release/6.2
ImageJ	NIH, USA	https://imagej.net
ZEN software	Carl Zeiss, Germany	www.zeiss.com/microscopy/en/products/software/zeiss-zen.html
MATLAB2024	MathWorks, USA	www.Mathworks.com
Paticle/Cell tracking (Matlab)	Zuo, Wenlong et al.^36^	https://github.com/JacobZuo/Tracking
SPSS Statistics 26	IBM, USA	www.ibm.com

### Resource availability

#### Lead contact

Further information and requests for resources and reagents should be directed to and will be fulfilled by the lead contact, Xiang Wang (wangxiang@cczu.edu.cn).

#### Materials availability

This study did not generate new unique reagents.

#### Data and code availability

• All data reported in this paper will be shared by the lead contact upon reasonable request.
• The original MATLAB program code is available in this paper’s supplemental information and is publicly accessible.
• Any additional information reported in this paper is available from the lead contact upon request.

### Experimental model and study participant details

#### Cell culture

NIH/3T3 fibroblast cells and primary rat airway smooth muscle cells were cultured in DMEM (Gibco, Thermo Fisher) supplemented with 10% FBS (Gibco). Cells were maintained in a humidified incubator with 5% CO_2_ at 37°C.

### Method details

#### Device fabrication

PDMS membranes were prepared by combining a 10:1 (w/w) mixture of Sylgard 184 elastomer and curing agent (Dow Corning, USA) in an 8 cm Petri dish. After spin-coating at 300, 200, and 100 rpm for 200, 60, and 60 s respectively (WH-SC-01, Wenhao Microfluidic Technology, China), a PDMS membrane with a thickness of 200 μm was formed. Two identical rectangular PDMS docking standers (5 × 5 × 10 mm) were positioned on the PDMS membrane with a square (10 × 10 mm) or an isosceles trapezoid (shorter base = 8, longer base = 10, height = 10 mm) region between them. After thermally curing at 80°C for 4 h, the two standers and the membrane between them were separated by cutting along the edges of the standers. To generate a uniformly stretched PDMS membrane, a U-shape 316 stainless steel wire (15, 16, or 17 mm width) was inserted into the two standers, generating 0%,10%, and 20% strain, respectively. A gradient-stretched membrane was produced by inserting the wire into the standers on a trapezoidal membrane until the membrane was stretched to a square shape (10 × 10 mm). To prevent torsional deformation of the prestrained membrane, the bottom of the standers was bound with a round glass coverslip after oxygen plasma treatment (PT-5S, Sanhe Boda Electromechanical Technology, China). Then, the device’s glass coverslip bottom was glued on the substrate of a 6-well culture plate using a thin layer of silicone grease (Baysilone, GE, USA) to avoid the device drifting during long-term imaging.

#### PDMS membrane functionalization

After thoroughly rinsing in 70% ethanol and drying, the upper membrane region of the device was functionalized to facilitate cell adhesion using a covalent bonding protocol. Briefly, after treating with oxygen plasma for 3 min, the membrane surface was incubated with 10% 3-aminopropyltriethoxysilane (APTES, Sigma-Aldrich, USA) for 2 h to silanate the hydroxyl groups. Following washing, it was treated with 2.5% glutaraldehyde (Aladdin, China) for 1 h at room temperature. Then, the activated region was coated with 0.1 mg/mL fibronectin (Thermo Fisher Scientific, USA) and incubated at 4°C overnight. Finally, the device was washed and immersed in PBS at 4°C, and used within 24 h.

#### Simulation and verification

The distribution of strain in prestrained PDMS membranes was analyzed using COMSOL Multiphysics 6.2 (COMSOL, Sweden). To simplify the modeling process, a two-dimensional model was established with a geometric scale matching physical dimensions (1:1). The PDMS film in the stretched region was defined as a linear elastic material with a density of 970 kg/m³, Young’s modulus of 10 kPa, and a Poisson’s ratio of 0.5. The initial boundary states on both sides of the film were set to 0. For the uniformly stretched PDMS membrane, boundary conditions were applied to induce a displacement of 1 mm in both positive and negative x-directions, corresponding to a 20% stretching of the PDMS membrane. For the gradient-stretched membrane, the two vertices on the longer base of the isosceles trapezoid were used as axes, with the diagonal sides rotating outward by one radian each, generating approximately 20% strain on the shorter base. The simulation objects were meshed using a free triangular mesh with a highly refined distribution, consisting of 3,244 (uniformly stretched) or 3,858 (gradient-stretched) triangular elements. The mesh size ranged from a minimum element size of 1.5 × 10^−3^ (uniformly stretched) or 1.2 × 10^−4^ mm (gradient-stretched) to a maximum of 0.4 mm (uniformly stretched) or 0.032 mm (gradient-stretched), with a curvature factor of 0.25. Strain percentages and elastic strain energy density (J/m^3^) were computed to assess the material’s response.

To validate the simulation result, polystyrene microspheres (20 μm in diameter, Sigma-Aldrich) were incorporated into uncured PDMS at an appropriate density, followed by the fabrication of PDMS membranes. For the uniformly stretched PDMS membrane, images of the central, border, and corner regions of the membrane were captured both before and after 20% stretch. For the gradient-stretched membrane, images of the central and two regions near the shorter and longer bases of the trapezoidal membrane were captured both before and after stretching. Then, these images were converted into binary format and exported into red (before stretching) and blue (after stretching) channels for comparison. The images from both channels were overlaid. The stretching’s effect was quantified by calculating the change in distance between two randomly chosen microspheres, compared to their initial separation, to evaluate the stretch degree at various membrane locations.

#### Measurement of Young’s modulus and surface tension of PDMS membranes

The PDMS membranes with different strains were measured for Young’s modulus using a NanoWizard 3 atomic force microscopy (JPK Instruments, Germany) in water at 22°C. A cantilever (k = 0.03 N/m, MLCT-O10, Bruker, Germany) glued with a spherical polystyrene bead (20 μm in diameter, Sigma-Aldrich) was used for the indentation experiment. Force-distance curves were collected on grids of 5 × 5 measurement points. Three to five grids located at the center of each membrane were sampled. The resulting force-distance curves were analyzed using a Hertzian model fit for the spherical indenter. The elastic modulus (E) was determined by the JPK data processing software.

For the measurement of contact angle, 5 μL water droplets were deposited on substrates using a 50 μL microsyringe (Hamilton, USA). After 30 s, the side view of droplets was captured photographically. More than 20 measurements were taken for each type of surface. The contact angles were analyzed using the drop analysis plug-in in ImageJ.

#### Cell culture and siRNA transfection

Cells were seeded at a density of 2 ×10^6^ cells/ml in a Petri dish 48 h before experiments. For cell culture on PDMS membranes, 8 mL of medium with cells (9620 cells/ml) was added to the well with the device inside, ensuring a density of 8000 cells/cm^2^ seeded on the substrate. Then the plate was carefully transferred to an incubator and allowed to stand for 2 h to facilitate cell sedimentation and attachment to the membrane. In siRNA inhibition experiments, pooled siRNAs targeting integrin and vinculin, along with a non-targeting control were used (RiboBio, China). A total of 80,000 cells were seeded into each well of a 6-well plate and transfected with 50 nM siRNA using Lipofectamine3000 in Opti-MEM (Gibco, Thermo Fisher). The medium was changed 8 h after transfection. The cells were seeded on a stretching device for live cell imaging or quantitative PCR analysis after 48 h.

#### Experimental setup and imaging analysis

The 6-well plate with each well containing the device was mounted onto a motorized stage (Prior Scientific, USA) of an Axio Observer 7 fluorescence microscope (Carl Zeiss, Germany) equipped with a 10×/0.25 Ph1 objective and an ORCA-Flash 4.0 camera (Hamamatsu, Japan) at 37°C and 5% CO_2_. Phase contrast multi-position live-cell imaging (30 min/frame) was recorded by the ZEN software (Carl Zeiss) 2 h after cell seeding.

For cell morphology analysis, cell edges were manually marked using ImageJ. Then the PAT-GEOM plug-in was used for image analysis. After applying the ellipse fitting function, parameters such as cell area and aspect ratio were quantified. The cell aspect ratio represents the ratio of the long axis to the short axis. For cell migration analysis, the positions of nuclei were manually marked with circles. The coordinate position and trajectory of cells were tracked according to the circular markers on each frame. Using a custom-written MATLAB program (MathWorks, USA) based on the Thiessen polygon principle, parameters such as cell migration speed, direction, and mean square displacement (MSD) were calculated based on the cell coordinate positions (Methods S1).

#### Immunofluorescence staining and quantification

3T3 cells on PDMS membranes were fixed with 4% paraformaldehyde (Sigma-Aldrich) for 30 min. Subsequently, cells were permeabilized with 0.1% Triton X-100 (Sigma-Aldrich) for 30 min and blocked with 1% bovine serum albumin (BSA, Roche, Swiss) for an additional 30 min. Afterward, primary antibodies including rabbit anti-myosin-X (HPA024223, Sigma-Aldrich), rabbit anti-integrin-β1 (ZRB1230, Sigma-Aldrich), or rabbit anti-vinculin (ab129002, Abcam, USA), were applied for incubation overnight at 4°C. Following this, samples were incubated with secondary antibody goat anti-rabbit AF-488 (Thermo Fisher) at room temperature for 2 h. The cytoskeleton F-actin was stained with FITC-conjugated phalloidin (Yeasen Biotech, China), and cell nuclei were stained with DAPI (Sigma-Aldrich). A confocal microscope (LSM710, Carl Zeiss) equipped with a 40×/0.75 NA objective or the Axio Observer 7 fluorescence microscope equipped with a 63×/1.4 NA objective was employed for imaging. To qualify the relative protein expression between experimental conditions, F-actin staining was used to define cell outlines and generate regions of interest (ROIs). The expression levels of integrin-β1 and vinculin were then quantified by measuring the mean intensity of immunofluorescence of each cell within ROIs.

#### Quantitative PCR analysis

The total mRNA expression levels of integrin-β1 and vinculin were measured by quantitative PCR (Q-PCR) with the associated primers (Table S1) (Sangon Biotech, China). Briefly, the total RNA of 3T3 cells was purified using an RNA isolation reagent (Vazyme, China). The extracted RNA was quantified using a Nanodrop 2000 Spectrophotometer (Thermo Fisher). For mRNA quantification, 500 ng total RNA was used to generate 1st strand cDNA using the reverse transcription kit (Vazyme). The reaction mixture (20 μL) contained 1 μL of cDNA in triplicates according to the manufacturer’s instructions. Q-PCR was performed using SYBR Green I (Vazyme) with a SteponePlus real-time PCR system (Applied Biosystems) at 95°C for 2 min, followed by 40 cycles of 95°C for 15 s and 60°C for 15 s. Melting curve analysis was performed at 95°C for 15 s, 60°C for 60 s, and 95°C for 15 s. The relative target mRNA expression to β-actin was determined using the 2^−ΔΔCT^ method. Fold changes in mRNA expression of genes were calculated as the ratio of experiment groups to the control groups from the resulting 2^−ΔΔCT^ values from four independent experiments.

#### Western blotting

3T3 cells were cultured for 72 h after transfection and lysed in RIPA lysis buffer (Beyotime, China) supplemented with protease and phosphatase inhibitor cocktails (Beyotime). The supernatant containing proteins was collected after centrifugation, and an appropriate amount of SDS-PAGE protein loading buffer (Beyotime) was added before boiling for 10 min. Electrophoresis was performed in an 8% Tris-Glycine gel apparatus, with 30 μL of sample loaded onto each lane for both control and experimental groups. 5 μL 180 kDa pre-stained protein marker (Vazyme) was also loaded. The voltage was set at 80 V, and electrophoresis was stopped when the pre-stained proteins were near the bottom of the gel. The transfer conditions included a constant current of 300 mA for 150 min. Subsequently, the membrane was blocked with 5% BSA at room temperature for 2 h, followed by overnight incubation at 4°C with primary antibodies against vinculin (ab129002, Abcam), integrin-β1 (ZRB1230, Sigma-Aldrich), and β-actin (Beyotime). The membrane was then incubated with secondary antibodies at room temperature for 1 h, and the bands were visualized by scanning using an Odyssey Sa Imager (LI-COR Biosciences, US).

#### Statistical analysis

For statistical analysis, a minimum of four independent experiments were performed. The data were analyzed using SPSS Statistics 26 (IBM, USA). Values are presented as means ± SEM, and error bars represent 95% confidence intervals. In the case of normally distributed data, the one-way ANOVA test was employed, with multiple comparisons adjusted using the Bonferroni method. In situations involving skewed distribution data, non-parametric testing, specifically the Kruskal-Wallis test, was used. The *p*-value was adjusted for multiple comparisons post the Wilcoxon Mann-Whitney test. Violin charts were used to depict data, showcasing the median as a central line and quartiles as lines on both sides (excluding extended head and tail portions).

## Supplemental information

Document S1. Figures S1–S3, Table S1 and Method S1
