# Supplementary material for: Focal adhesion-mediated directional cell migration guided by gradient-stretched substrate
Source: iScience. 2024 Jul 4;27(8):110446. doi: 10.1016/j.isci.2024.110446 (PMC11301061; doi:10.1016/j.isci.2024.110446)
Supplement: Document S1. Figures S1–S3, Table S1 and Method S1 [file mmc1.pdf]

iScience, Volume *27*

Supplemental information

Focal adhesion-mediated directional cell migration guided by gradient-stretched substrate

Zijia Chen, Xiaoning Han, Bo Che, Huiping Feng, Yue Zhou, Linhong Deng, and Xiang Wang

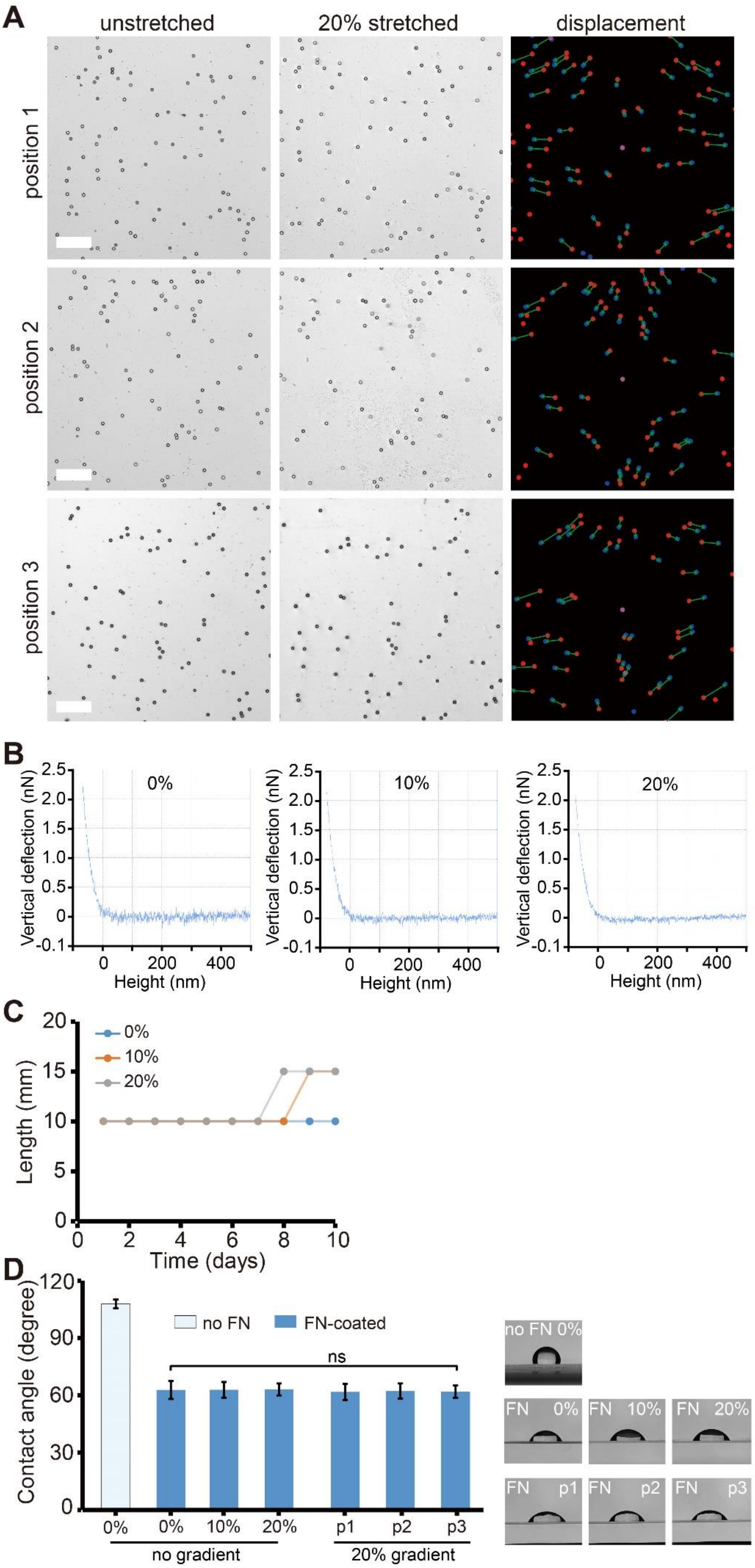

A
unstretched
20% stretched
displacement
position 1
position 2
position 3
B
0%
10%
20%
Vertical deflection (nN)
Height (nm)
C
0%
10%
20%
Length (mm)
Time (days)
D
Contact angle (degree)
no FN
FN-coated
ns
no gradient
20% gradient
p1
p2
p3
no FN 0%
FN 0%
FN 10%
FN 20%
FN p1
FN p2
FN p3

Figure S1. Characterization of PDMS membranes, related to Figure 1. (A) Displacement of microbeads embedded in membranes prestrained to 20%. Images of microbeads at three positions, as referenced in Fig. 1C, were captured before and after stretching. The displacement paths of microbeads are illustrated with arrows in the left column. Bars = 200 μm. (B) Representative force-distance curves obtained by AFM-based force spectroscopy for measuring Young's modulus of PDMS membranes under prestrain conditions of 0, 10%, and 20%. (C) Recovery behavior of PDMS membrane strain under different static stretch conditions. The length of the PDMS membranes, initially set at 10 mm, was measured following the release of stretching forces at specified time points (n = 3 independent experiments). (D) Contact angles of native PDMS membranes and membranes coated with fibronectin under different strain conditions (n = 3 independent experiments). Data are presented as means ± SEM. One-way ANOVA test, ns = non-significant.

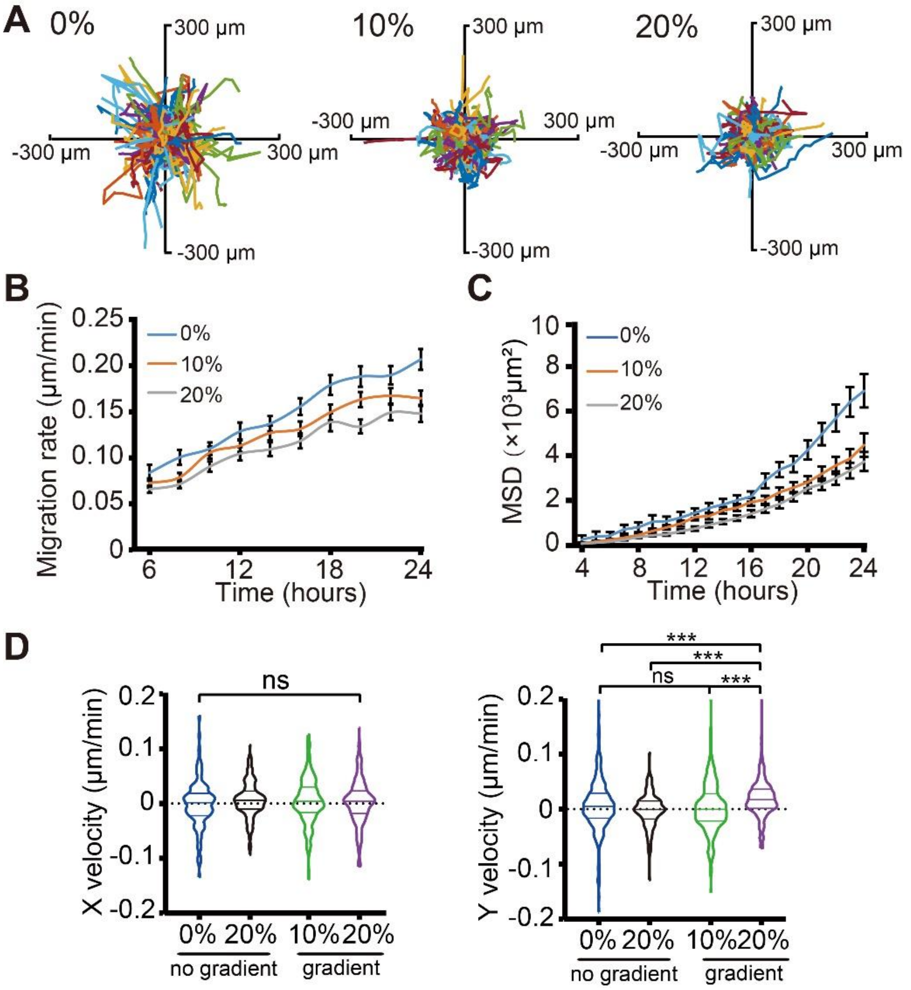

Figure S2. Impact of prestrained substrates on ASM cell migration, related to Figures 4 and 5. (A) Trajectories of randomly selected migrating ASM cells on different prestrained substrates over 24 hours. (B) Migration rates of ASM cells cultured on different prestrained substrates (n = 4 independent experiments). Data are presented as means ± SEM. (C) Mean squared displacement (MSD) plotted versus time lag for cells on different prestrained conditions (n = 4 independent experiments). Data are presented as means ± SEM. (D) X and Y velocity of ASM cells on normal prestrained (0, 20%) and gradient

prestrained (10%, 20%) substrates (n > 200 cells). Kruskal-Wallis test, ns = non-significant, *** $p <$ 0.001.

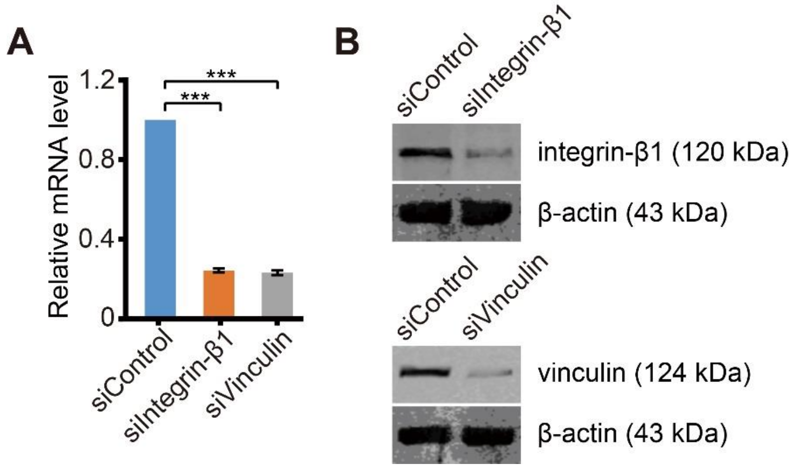

Figure S3. siRNA knockdown of integrin-β1 and vinculin in 3T3 cells, related to Figure 5. (A) Q-PCR analysis of siRNA knockdown of integrin-β1 and vinculin (n = 4 independent experiments). Data are presented as means ± SEM. One-way ANOVA test, *** $p < 0.001$. (B) Western blot for siRNA knockdown of integrin-β1 and vinculin. β-actin is the respective loading control.

Table S1 Primers used in the quantitative PCR analysis, related to Figure 3

Gene	Sequence
β-actin F	AGGGAAATCGTGCGTG
β-actin R	AACCGCTCGTTGCCAA
integrin-β1 F	GAGGTTCAATTTGAAATTAGC
integrin-β1 R	GGCTCTGCACTGAACACATTC
vinculin F	ATGACATCCTCCGCTCCCTTGG
vinculin R	TGCCGTCGCCACTTGTTTAGC

Methods S1: Original MATLAB program code, related to Figures 4 and 5, and Figure S2

```
SamplePath2 = 'C:\Users\Administrator\Desktop\processed-P2-03\';
fileExt2 = '*.tif';
files2 = dir(fullfile(SamplePath2,fileExt2));

SamplePath1 = 'C:\Users\Administrator\Desktop\P2-03\';
fileExt1 = '*.tif';
```

```
files1 = dir(fullfile(SamplePath1,fileExt1));

% Traverse each image in the path, the cell nucleus is marked as white 255, and the rest of the positions are collectively referred to as the background. Randomly generate values below 100 as pixel values
len1 = size(files1,1);
for m=1:len1;
    fileName1 = strcat(SamplePath1,files1(m).name);
    f=imread(fileName1);
    fr=f(:,:,1);
    [x, y] = size(fr);
     for i=1:1:x
          for j=1:1:y
               if fr(i, j) == 0
                    g(i, j) = 255;
               else
                    g(i, j) = round(rand(1,1)*100);
               end
          end
     end
     g0=mat2gray(g);
     imwrite(g0,strcat(SamplePath2,files1(m).name,'.tiff'));
end


% run main program Paticle/Cell tracking
[Trace_All, ImageInfo] = Tracking('C:\Users\Administrator\Desktop\processed-P2-03.tif', 'AutoThreshold', 'on');

% location
n=length(Trace_All);
for i=1:1:n
     a=length(Trace_All{1,i});
     if a ~= 22
         Trace_All{1,i}=[];
     end
end
Trace_All(cellfun(@isempty,Trace_All))=[];

n=length(Trace_All);
for i=1:1:n
     coordinate(2*i-1,:) = Trace_All{1,i}(:,2);
     coordinate(2*i,:) = Trace_All{1,i}(:,3);
end
```

```
% migration speed
n=length(coordinate)/2;
for i=1:1:n
    for j=1:1:21
        x1=coordinate(2*i-1,j+1);x0=coordinate(2*i-1,j);
        y1=coordinate(2*i,j+1);y0=coordinate(2*i,j);
        a=abs(x1-x0)*0.632;
        c=abs(y1-y0)*0.632;
        b=(a^2+c^2)^0.5;
        speed(i,j) = b/60;
    end
end

% MSD
n=length(coordinate)/2;
for i=1:1:n
    for j=1:1:21
        x1=coordinate(2*i-1,j+1);x0=coordinate(2*i-1,1);
        y1=coordinate(2*i,j+1);y0=coordinate(2*i,1);
        a=abs(x1-x0)*0.632;
        c=abs(y1-y0)*0.632;
        b=(a^2+c^2)^0.5;
        MSD(i,j) = b^2;
    end
end

% migration direction
A = coordinate.';
a=[];c=[];
n=length(coordinate)/2;
for i=1:1:n
        x3=A(1,2*i-1);y3=A(1,2*i);
        x24=A(22,2*i-1);y24=A(22,2*i);
        a(i,4)=(x24-x3)*0.632;c(i,4)=(y3-y24)*0.632;
end
cc=c/1260;%21*60=1260min
aa=a/1260;
```
